# Synthesis, Biological, and Computational Evaluations of Conformationally Restricted NAD-Mimics as Discriminant Inhibitors of Human NMN-Adenylyltransferase Isozymes

**DOI:** 10.3390/ph17060739

**Published:** 2024-06-06

**Authors:** Federica Matteucci, Marta Ferrati, Eleonora Spinozzi, Alessia Piergentili, Fabio Del Bello, Gianfabio Giorgioni, Leonardo Sorci, Riccardo Petrelli, Loredana Cappellacci

**Affiliations:** 1 Medicinal Chemistry Unit, School of Pharmacy, Chemistry Interdisciplinary Project (ChIP), University of Camerino, Via Madonna delle Carceri, 62032 Camerino, Italy; federica.matteucci@unicam.it (F.M.); marta.ferrati@unicam.it (M.F.); eleonora.spinozzi@unicam.it (E.S.); alessia.piergentili@unicam.it (A.P.); fabio.delbello@unicam.it (F.D.B.); gianfabio.giorgioni@unicam.it (G.G.); loredana.cappellacci@unicam.it (L.C.); 2 Division of Bioinformatics and Biochemistry, Department of Science and Engineering of Matter, Environment and Urban Planning (SIMAU), Polytechnic University of Marche, 60131 Ancona, Italy

**Keywords:** NAD metabolism, anticancer therapy, NAD analogues, NMNAT inhibitors, inhibitor design, dinucleotides

## Abstract

Nicotinamide adenine dinucleotide (NAD) cofactor metabolism plays a significant role in cancer development. Tumor cells have an increased demand for NAD and ATP to support rapid growth and proliferation. Limiting the amount of available NAD by targeting critical NAD biosynthesis enzymes has emerged as a promising anticancer therapeutic approach. In mammals, the enzyme nicotinamide/nicotinic acid adenylyltransferase (NMNAT) catalyzes a crucial downstream reaction for all known NAD synthesis routes. Novel nicotinamide/nicotinic acid adenine dinucleotide (NAD/NaAD) analogues **1**–**4**, containing a methyl group at the ribose 2′-*C* and 3′-*C*-position of the adenosine moiety, were synthesized as inhibitors of the three isoforms of human NMN-adenylyltransferase, named *h*NMNAT-1, *h*NMNAT-2, and *h*NMNAT-3. An NMR-based conformational analysis suggests that individual NAD-analogues (**1**–**4**) have distinct conformational preferences. Biological evaluation of dinucleotides **1**–**4** as inhibitors of *h*NMNAT isoforms revealed structural relationships between different conformations (North-*anti* and South-*syn*) and enzyme-inhibitory activity. Among the new series of NAD analogues synthesized and tested, the 2′-*C*-methyl-NAD analogue **1** (*K*_i_ = 15 and 21 µM towards NMN and ATP, respectively) emerged as the most potent and selective inhibitor of *h*NMNAT-2 reported so far. Finally, we rationalized the in vitro bioactivity and selectivity of methylated NAD analogues with in silico studies, helping to lay the groundwork for rational scaffold optimization.

## 1. Introduction

Tumor cells have unique metabolic adaptations that facilitate rapid growth and proliferation. A hallmark of this altered metabolism is the heavy reliance on glycolysis for energy production (known as the Warburg effect), making them more vulnerable to the disruption of glucose energy metabolism. While overall less efficient than aerobic respiration in terms of energy yield (2 versus 32 adenosine triphosphate (ATP) molecules), anaerobic glycolysis is advantageous for cancer cell proliferation [1]. First, not involving the additional steps of the Krebs cycle and oxidative phosphorylation, anaerobic glycolysis allows for faster ATP production, thus partially compensating for the lower ATP yield. Another advantage of shifting to anaerobic glycolysis is the concurrent production of metabolic intermediates that can be used as building blocks to fuel cancer proliferation [2]. To this end, glucose 6-phosphate (G6P) and 3-phosphoglycerate (3-PGA) are critical intermediates. G6P is diverted to the pentose phosphate pathway for the biosynthesis of nucleotides to support DNA and RNA production [3], while 3-PGA can be directed to the synthesis of serine, which, in turn, can support the production of other amino acids, lipids, and folic acid [4]. In addition, the preference for anaerobic glycolysis limits the production of reactive oxygen species, mainly generated through the electron transport chain, further promoting cancer cell proliferation [1]. Nicotinamide adenine dinucleotide (NAD) is an essential coenzyme governing multiple metabolic pathways, including glycolysis and serine biosynthesis [5,6]. The pivotal function of NAD in the metabolic processes of cancer cells has opened up new avenues for anticancer therapies aiming at limiting intracellular NAD content. This could involve inhibiting NAD biosynthesis, boosting NAD degradation, or both [7,8,9]. Among viable NAD biosynthetic target enzymes, nicotinamide mononucleotide adenylyltransferase (NMNAT) is relatively unexplored. The enzyme NMNAT converts ATP and the nicotinamide mononucleotide (NMN) or its deamido form (NaMN) into diphosphate (PPi) and NAD or nicotinate adenine dinucleotide (NaAD), respectively. Then, NaAD becomes NAD through the activity of NAD synthetase. This dual-substrate specificity confers to NMNAT a central role in NAD biosynthesis, catalyzing downstream reactions to all known synthetic routes [10]. One of the challenges in targeting human NMNAT in mammals for cancer treatment is the existence of three NMNAT isoforms, namely *h*NMNAT-1, *h*NMNAT-2, and *h*NMNAT-3, with distinct tissue distribution, subcellular localization, and catalytic properties [11]. *h*NMNAT-1 is the most abundant and ubiquitously expressed isoform across various tissues, with a predominant localization within the cell nucleus. Notably, it exhibits the highest catalytic efficiency among the three isoforms. In contrast, *h*NMNAT-2 shows a specific localization into the brain, localized on the Golgi apparatus’s cytosolic surface. Finally, *h*NMNAT-3, although also ubiquitously expressed, predominantly resides within the mitochondrial matrix, lysosomes, and cytosol.

In addition to the overall theorized advantage of disrupting NAD metabolism in cancer cells, targeting specific isoforms of NMNAT can prove effective in treating certain types of cancer or overcoming cancer resistance.

For instance, NMNAT plays a crucial role in activating the prodrug tiazofurin by converting its derivative, tiazofurin 5′-monophosphate (TrMP), into the antineoplastic agent tiazofurin adenine dinucleotide (TAD). Our findings indicated that this conversion is primarily catalyzed by *h*NMNAT-1 and, less efficiently, by *h*NMNAT-3 [12]. Notably, a common trait observed in TAD-resistant tumor cells is the significant reduction of NMNAT activity, by up to 95% [13], rendering them potentially more susceptible to *h*NMNAT-1 inhibition. Conversely, *h*NMNAT-2 is the isoform involved in the activation of Vacor, an old rat poison that has shown toxicity to *h*NMNAT-2-expressing cancer cells [14]. *h*NMNAT-2 is also highly expressed in a variety of solid tumors like colorectal, lung, and ovarian cancers, where it plays a significant role in tumor development. Studies have shown that *h*NMNAT-2 is upregulated in colorectal cancer (CRC), and it participates in the tumorigenesis of CRC in a p53-dependent manner, thus making *h*NMNAT-2 a promising target for diagnosis and treatment of CRC [15].

Despite the pivotal role of NAD in cancer development, 20% of lung, renal, and colorectal cancers have a reduced expression of *h*NMNAT-1, sometimes accompanied by a significant decrease in NAD content [16,17,18]. In the complexity of tumor cell metabolism, this appears to be a compromise that overall favors tumor progression and chemoresistance but, at the same time, offers *h*NMNAT-1 as a vulnerable target to induce lethality in cancer cells, with limited toxicity to normal cell lines.

Although the available findings clearly point to *h*NMNATs, and more precisely to *h*NMNAT-1 and *h*NMNAT-2, as promising targets in specific cancer types, only a few inhibitors have been identified and characterized to date. Gallotannin, a polyphenolic plant metabolite with various targets, non-specifically inhibits all three isoforms, with *h*NMNAT-3 being the most sensitive (IC_50_ = 2 μM) [11,19]. Nucleotide polyphosphates, namely Np_3_AD, Np_4_AD, and Nap_4_AD, showed selective inhibition against the different isoforms, although with IC_50_ values in the micromolar range [20,21].

In the present study, we designed, synthesized, and biologically evaluated a novel series of 2′- and 3′-*C*-methyl-substituted NAD analogues as inhibitors of *h*NMNAT isoforms.

## 2. Results and Discussion

### 2.1. Chemistry

**Scheme 1:**
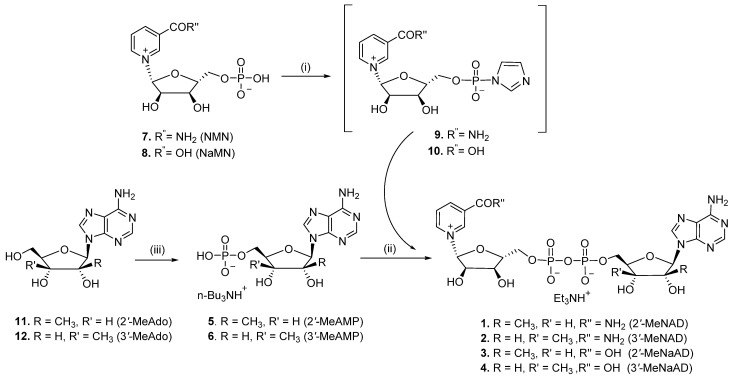
Synthesis of NAD analogues **1**–**4**. Reagents and conditions: (i) CDI, DMF, rt; (ii) DMF, rt; (iii) POCl_3_, (CH_3_O)_3_PO, 0 °C; then (*n*-Bu)_3_N, DMF.

The synthesis of 2′-MeNAD (**1**), 3′-MeNAD (**2**), 2′-MeNaAD (**3**), and 3′-MeNaAD (**4**) was performed following the procedure reported in Scheme 1. The electrophilic nicotinamide riboside monophosphate imidazolide (**9**) and nicotinic acid riboside monophosphate imidazolide (**10**) were prepared by reaction of the commercially available nicotinamide riboside 5′-monophosphate (NMN, **7**) or nicotinic acid riboside 5′-monophosphate (NaMN, **8**) with 1,1′-carbonyldiimidazole (CDI) as activating agent. The formation of imidazolides **9** or **10** was monitored by high performance liquid chromatography (HPLC) and required no isolation. Coupling of mono *n*-tributylammonium salt of the 2′-*C*-methyl-adenosine 5′-monophosphate (2′-MeAMP, **5**) or 3′-*C*-methyl-adenosine 5′-monophosphate (3′-MeAMP, **6**) with imidazolides **9** or **10** (molar ratio 1.5:1) gave the desired dinucleotides **1**–**4**, which were purified by chromatography on a DEAE Sephadex (HCO_3_^−^ form) column eluting with a linear gradient of H_2_O and 0.5 M triethylammonium bicarbonate (TEAB) and characterized as triethylammonium salts by high-resolution ^1^H-NMR, ^31^P-NMR spectroscopy, and mass spectrometry (API-ESI). 2′-MeAMP (**5**) and 3′-MeAMP (**6**) were obtained by phosphorylation of the nucleoside analogues 2′-MeAdo (**11**) [22] and 3′-MeAdo (**12**) [23], respectively, by treatment with trimethyl phosphate and POCl_3_, following the procedure reported by Yoshikawa and colleagues [24]. They were purified by chromatography on a DEAE Sephadex (HCO_3_^−^ form) column and then treated with Dowex 50 *×* 8 (H^+^ form) to obtain the corresponding free acids, which were converted into the *n*-tributylammonium salts **5** and **6** by reaction with tributylamine in *N*,*N*-dimethylformamide (DMF).

### 2.2. Conformational Analysis

**Figure 1:**
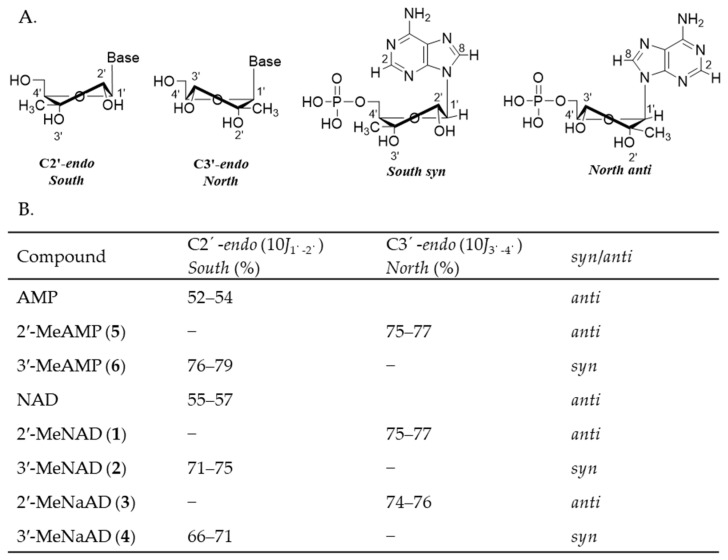
Conformational analysis of AMP, NAD, and 2′-, and 3′-AMP/NAD analogues. (**A**) Major conformations of furanose (*North*/*South*) and adenine base (*syn*/*anti*) in 2′- or 3′-substituted adenine nucleosides/tides. (**B**) Percentage of adopted conformations.

We next carried out a conformational analysis to further characterize this series of methylated compounds. Conformational studies of nucleosides and nucleotides in solution indicated that the furanose generally exists in a conformational equilibrium between the C2′-endo (South-type) and C3′-endo (North-type) form, and interconversion is rapid on a ^1^H-NMR time scale [25]. On the other hand, the nucleobase is oriented perpendicularly to the plane of the ribose ring and can assume two different conformations, by rotation about the N-glycosidic bond: in the syn conformation, the nucleobase points towards the ribose, and in the anti conformation it is away from the sugar [26]. The preferred conformation of a given adenine nucleoside or nucleotide can be extracted from ^1^H-NMR data. For conformationally restricted nucleosides and nucleotides, the magnitude of the H-1′/H-2′ and H-3′/H-4′ coupling constants is directly correlated to the ratio of the C2′-endo/C3′-endo conformers. Altona et al. showed that the relative percentage of the C2′-endo/C3′-endo population can be estimated by taking 10J_1′–2′_ or 10J_3′–4′_ [25]. In addition, the preference for syn or anti conformations can be deduced experimentally by nuclear Overhauser effect (NOE) experiments. Using Altona’s approach, we determined the preferred conformations for all the newly substituted adenosine monophosphate (AMP) (**5**, **6**) and NAD analogues (**1**–**4**). It is known that natural AMP and NAD predominantly adopt the anti conformations for the adenine base and the South-type sugar puckering for the ribose [27]. The large J_3′–4′_ coupling constants (~7.4–7.7 Hz) detected for compounds **1** and **3** are strongly suggestive of adopted C3′-endo (North-type) conformations, in contrast with C2′-endo (South-type) conformations that usually exhibit significantly smaller J_3′–4′_ values (~2.0 Hz). On the other hand, the large J_1_*_′_*_–2_*_′_* coupling constant values (~6.6–7.5 Hz) detected for compounds **2** and **4** suggested that they predominantly populate the C2′-endo (South-type) conformation. The outcomes of our analysis are depicted in Figure 1.

The glycosidic bond conformations of AMP/NAD conformationally-restricted analogues have not previously been investigated systematically. NOE experiments with irradiation of H-1′ of 2′-*C*-methyl-adenosine 5′-monophosphate (2′-MeAMP, **5**) showed the complete lack of H-8 enhancement in the purine ring, supporting a spatial arrangement where H-8 and H-1′ are not proximate, as would be the case in the anti conformer. On the contrary, irradiation of H-1′ of 3′-*C*-methyl-adenosine 5′-monophosphate (3′-MeAMP, **6**) showed the complete lack of H-2 enhancement in the purine ring, as would be the case in the syn conformer (Figure 1).

Our NOE data show a similar trend for the H-1′ enhancement values in the NAD series (**1–4**) as in the AMP series (**5**, **6**). Therefore, it seems reasonable to propose that the conformations of glycosyl bonds are unaffected by pyrophosphate bond formation. Consequently, 2′-MeNAD (**1**) and 2′-MeNaAD (**3**) showed a clear preference for the anti conformation and a sugar puckering in *North*-type conformation. In contrast, both 3′-MeNAD (**2**) and 3′-MeNaAD (**4**) should be predominantly syn and in *South*-type conformation. 

### 2.3. Biological Evaluation

In order to assess the bioactivity of the novel conformationally restricted NAD analogues, compounds **1**–**4** were evaluated as potential inhibitors of the three human NMNAT isoforms. Initially, we performed a screening at a fixed concentration of 0.5 mM of all derivatives, which included, in addition to the NAD analogues (**1**–**4**), the methylated adenosines (**11**, **12**) and adenosine monophosphates (**5**, **6**) as well. While we did not observe any appreciable inhibition with the adenosine and AMP scaffold and the 3′-methylated NAD analogues, the 2′-methylated NAD derivatives consistently exhibited an enzyme inhibition of over 50%. Such NAD prototypes offer the advantage of simultaneously occupying both substrate subsites (NMN and ATP) within the active sites of enzymes displaying sequential ternary complex kinetic mechanisms. Since NMNAT catalyses its reaction via ternary complex formation (E-NMN-ATP), the enzyme should be effectively and specifically inhibited by conformationally restricted NAD analogues possessing the features of NMN and AMP. We argue that the absence of inhibition of the smaller methylated nucleosides (**11**, **12**) and nucleotides (**5**, **6**) can be attributed to their inability to engage both NMN and AMP subsites.

**Figure 2:**
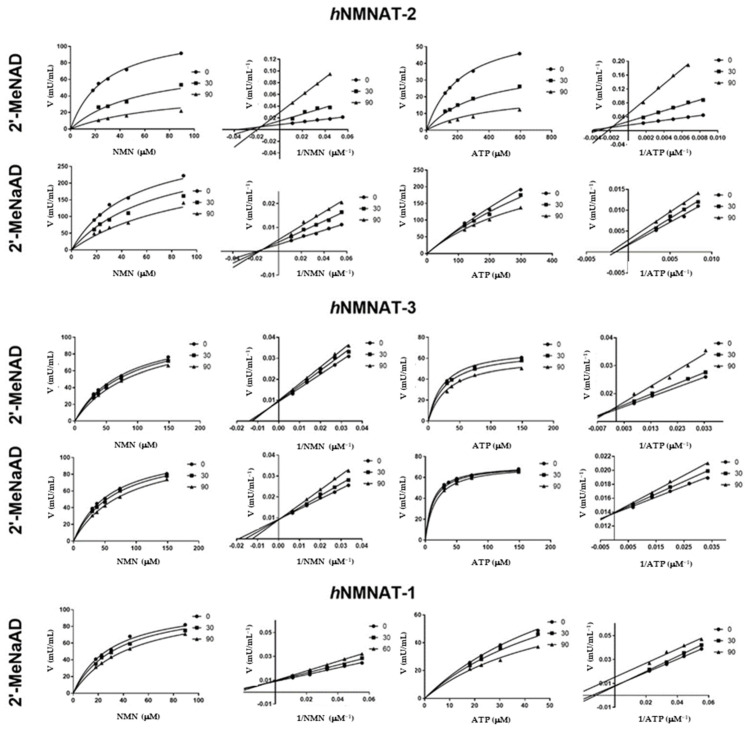
Human NMNAT inhibition by 2′-MeNAD (**1**) and 2′-MeNaAD (**3**). Hyperbolic plots of initial rates as a function of NMN or ATP concentration measured at a fixed saturating concentration of one substrate (0.5 mM) in the absence and presence of varying concentrations of 2′-MeNAD and 2′-MeNaAD. The same data are also presented by a double-reciprocal (Lineweaver–Burk) plot illustrating different modes of inhibition. Each data point is the average value derived from three replicates. Error bars are explicitly omitted for clarity.

Next, a detailed kinetic characterization was performed to determine inhibitory constants and mode of inhibition of 2′-methyl NAD analogues. Table 1 and Figure 2 indicate varying potency of our compounds against the three isozymes. While *h*NMNAT-1 and -3 are inhibited in the high micromolar range (~100–300 µM), *h*NMNAT-2 is potently and selectively inhibited in the low micromolar range (~15–50 µM). In line with our chemical design rationale, *K*_i_ is consistently similar for NMN and ATP within each enzyme, supporting the concurrent inhibition of NMN and ATP subsites. The deamidated version of our 2′-methyl derivatives follows the same isozyme-specificity trend of 2′-MeNAD, underlining that the modified adenosyl moiety triggers the enzyme perturbation effect.

### 2.4. Mechanistic Analysis of hNMNAT Inhibition by 2′-MeNAD and 2′-MeNaAD

As expected with NAD analogues, the steady-state kinetic analysis of compounds **1** and **3** disclosed a pronounced competitive component for both NMN and ATP, particularly accentuated in the case of *h*NMNAT-3 (Figure 2). Notably, for *h*NMNAT-2, a mixed-type inhibition pattern was observed, hinting at a binding site that only partially overlaps with the enzyme active site. This outcome provides insight into the high potency and specificity of 2′-MeNAD (**1**) for *h*NMNAT-2 and underscores the necessity for in-depth investigation of the inhibitor binding site, as elaborated in the subsequent section. As an exception, 2′-MeNaAD (**3**) showed an uncompetitive character versus ATP for *h*NMNAT-1 and *h*NMNAT-2 but not for *h*NMNAT-3. Although intriguing, we did not deem it necessary to further investigate such an inhibitory mechanism due to its modest potency. Instead, we focused on elucidating the binding mode of the most potent *h*NMNAT-2 inhibitor, 2′-MeNAD (**1**). This seemed particularly interesting as the steady-state kinetic analysis revealed a mixed-type inhibition with a strong non-competitive character.

### 2.5. The Structural Rationale of 2′-Methylated NAD Analogues’ Bioactivity

The biological evaluation of conformationally restricted NAD analogues against human NMNAT isoforms indicated that only the 2′-methyl-substituted NAD (i.e., with predominant North-type conformation) exerted a marked inhibition. In contrast, the 3′-methyl-substituted NAD (with a favored South-type conformation) did not affect the enzyme activity. In order to rationalize such selectivity, it is important first to highlight the conformational properties of the “natural” NAD bound to NMNAT enzymes and the typical ligand-pocket interactions. Upon examining about a dozen structures of the NMNAT family (including bacterial and archaeal members) with bound NAD, we can conclude that NMNATs consistently accommodate nucleotides with the South-type form of ribose, as well as being typically observed in proteins with a Rossman fold.

**Figure 3:**
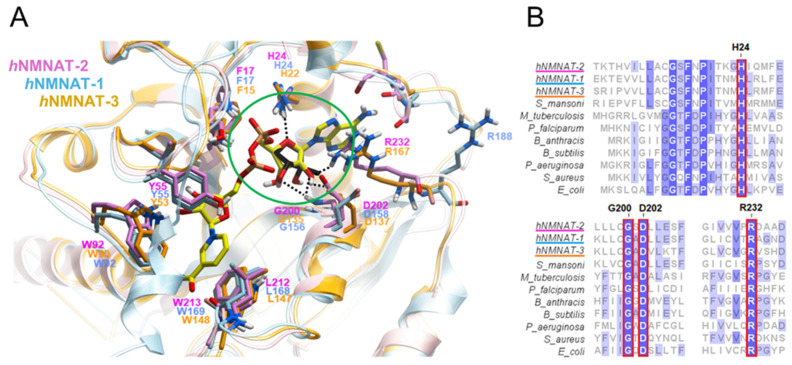
NAD binding across *h*NMNATs. (**A**) Superposition of *h*NMNATs in complex with NAD product showing the native *South*-type conformation of adenosyl ribose and its stabilizing interactions. PDB IDs for *h*NMNAT-1 and *h*NMNAT-3 are 1kqn and 1nuu, respectively. For *h*NMNAT-2, we generated a model with Alphafold2. Key residues of the NAD binding site and the NAD molecule (in yellow) are rendered as sticks. Dashed lines represent the H-bonds. Inside the green circle, the *North*-type conformation of a 2′-methyl-ribose (in black) is superposed on native adenosyl ribose, showing its perturbation effect. (**B**) Excerpt of a structure-based sequence alignment of representative NMNATs, including eukaryotic and prokaryotic pathogens. The intensity of the background color indicates sequence conservation. Key residues (numbered by *h*NMNAT-2 sequence) involved in adenosine binding are outlined with red boxes.

Figure 3A shows several interactions with conserved aminoacids (Figure 3B) in the nucleotide-binding site to stabilize such a conformation. For example, a conserved glycine (e.g., G200 in NMNAT-2) typically engages both the 3′- and 2′-OH groups with H-bonds, while the 2′-OH additionally forms two H-bonds with an aspartate (D202) and arginine (R232) (Figure 3A). In this conformation of the adenosine ribose, the hydrogen positioned at 3′ points toward a largely unobstructed space, thus possibly explaining the lack of effect of the 3′-methyl substitution. 

**Figure 4:**
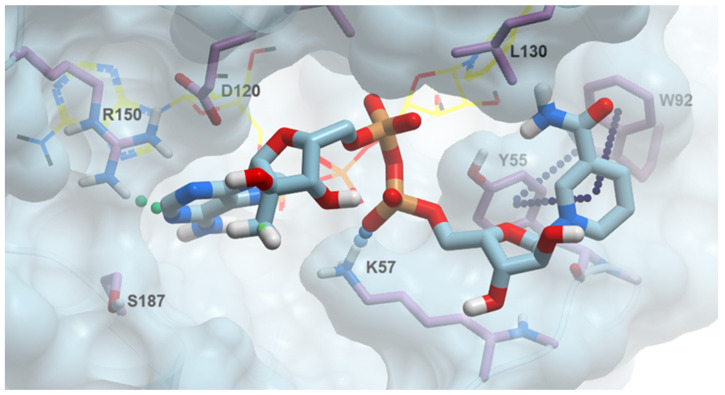
2′-MeNAD (**1**) docking pose in *h*NMNAT-2. H-bonds and salt-bridges are shown as colored spheres, while π-interactions are in black. 2′-MeNAD is in stick representation, while the position of the natural NAD product is presented in light yellow wires. Residues that are likely to interact with the 2′-MeNAD are displayed.

In contrast, the North-type conformation of the 2′-methyl-NAD, as modelled in Figure 3A, would not preserve the natural interactions of the ribose with the binding pocket: the 3′-OH would be too far away to maintain its contact with the glycine, while 2′-OH retains only one H-bond out of three. Finally, the hydrophobic 2′-methyl group would face two charged aminoacids, thus destabilizing the H-bonding network around the ribose. Consequently, we propose that the 2′-methyl-substituted NAD, not fitting the traditional AMP subsite, should bind differently (see Figure 4), in line with its predominant noncompetitive character. It is worth noting that the adenosyl moiety binding site is universally conserved across the NMNAT family (Figure 3B), which includes actively pursued targets against tuberculosis [28,29,30] and other bacterial pathogens [31,32], the protozoan malaria parasite [33], and the flatworm Schistosoma mansoni [34]. This observation expands future potential applications of 2′-methyl-NAD analogues to other clinically relevant members of the NMNAT family, with minimal impact on normal human host cells.

### 2.6. 2′-MeNAD Binding Mode

To assess the 2′-MeNAD (**1**) binding mode and to obtain a basis for rational improvement of our best scaffold, we performed an accurate in silico binding analysis of **1** on *h*NMNAT-2 (Figure 4). Without an available 3D structure, we resorted to a high-quality 3D model obtained with Alphafold2 as the template for a dynamic docking approach, where all major conserved aminoacids of the active site were set as flexible. The best pose is illustrated in Figure 4 and compared to the native NAD binding pose. Interestingly, while remaining in the larger active site binding area, the NAD methyl analogue does not overlap with native NAD (with an average shift of about 10–15 Å). The molecule has three major anchor points (Figure 4). At the adenosyl site, the adenine contacts Arg150 with an edge-face Pi-cation interaction and an H-bond with the N1 atom. Asp120 further stabilizes the adenine orientation with a Pi-anion interaction. A salt-bridge is present between Lys57 and the nicotinosyl phosphate, while the nicotinate ring is mainly oriented through two offsets π-interactions with both Tyr55 and Trp92.

## 3. Materials and Methods

### 3.1. Chemistry

#### 3.1.1. General Procedure

All commercial reagents and solvents were purchased from Merck (Darmstadt, Germany) and were used as provided unless otherwise indicated. Solvents were dispensed under argon. The analytical samples of nucleotides were lyophilized or dried in vacuo over P_2_O_5_. Thin layer chromatography (TLC) was run on silica gel 60 F_254_ plates and silica gel RP-18m plates with the indicated solvent system; silica gel 60 (70–230 mesh, Merck) and DEAE Sephadex for column chromatography were employed. Analytical HPLC (Agilent 1100 series, Agilent Technologies, Santa Clara, CA, USA), coupled with a photodiode array detector (DAD), was performed on a Varian Microsorb column (C18, 5 μm, 4.6 × 250 mm) with a 0.5 mL/min flow rate. An isocratic or linear gradient of 0.04M TEAB and aqueous MeCN (70%) was used. Nuclear magnetic resonance spectra were recorded with a Bruker 500 Ascend (Bruker BioSpin Corporation, Billerica, MA, USA) with Me_4_Si (TMS), as the internal standard for ^1^H-NMR, and external H_3_PO_4_ for ^31^P. Chemical shifts are reported in part per million (*δ*), and coupling constants, when provided, are expressed in hertz (Hz). Values given for coupling constants are first order. Stationary NOE experiments were run on degassed solutions at 25 °C with saturation of individual lines within the multiplets and by internal subtraction of on- and off-resonance spectra. 1D NOE experiments were acquired with a 500 ms mixing time and a 2.5 s acquisition time. High-resolution mass spectra were recorded on an Agilent TOF II TOF/MS instrument equipped with an ESI or APCI interface.

#### 3.1.2. P^1^-[5′-(2′-*C*-Methyl-β-D-ribofuranosyl)adenine]-P^2^-[5′-(β-D-ribofuranosyl) nicotinamide] Pyrophosphate (Triethylammonium Salt) (2′-MeNAD, **1**)

1,1′-Carbonyldiimidazole (CDI) (220 mg, 1.36 mmol) was added to a suspension of **7** (56.8 mg, 0.17 mmol) in dry DMF (2.5 mL) under nitrogen atmosphere. The reaction mixture was stirred at room temperature for 4 h. A ^31^P-NMR spectrum indicated that all starting NMN had been consumed, and a new peak at −10.4 ppm was formed. The reaction was stopped by adding dry methanol (103 μL) to hydrolyze excess CDI, and the solution was stirred at room temperature for an additional 30 min. To the resulting imidazolide **9**, a solution of 9*H*-(2′-*C*-methyl-β-D-ribofuranosyl)adenine-5′-monophosphate tributyl-ammonium salt (**5**, 97.5 mg, 0.27 mmol) in dry DMF (2 mL) was added. The reaction mixture was kept at 30 °C, and the reaction progress was monitored by ^31^P-NMR. After 3 days, water (3 mL) was added, and the mixture was washed with CHCl_3_ (2 × 10 mL) and Et_2_O (2 × 10 mL). The crude residue was purified by chromatography on a DEAE Sephadex (HCO_3_^−^ form) column, eluting with a linear gradient of H_2_O and 0.5 M TEAB, giving **1** as white foam after lyophilization (18 mg, 16% yield, triethylammonium salt). The purity of **1** was 98% (t_R_ = 6.5 min) as determined by analytical HPLC.

^1^H-NMR (D_2_O, 500 MHz): *δ* 0.76 (s, 3H, CH_3_), 1.25 (t, *J* = 7.4 Hz, 9H, (C*H_3_*CH_2_)_3_NH^+^), 3.17 (q, *J* = 7.2 Hz, 6H, (CH_3_C*H_2_*)_3_NH^+^), 4.01–4.11 (m, 2H, H5′), 4.13–4.21 (m, 2H, H5′′), 4.27 (pseudo q, 2H, H4′, H4′′), 4.31 (d, *J* = 7.7 Hz, 1H, H3′), 4.39 (t, *J* = 6.8 Hz, 1H, H3′′), 4.51 (pseudo t, 1H, H2′′), 5.84 (s, 1H, H1′), 5.91 (d, *J* = 5.13 Hz, 1H, H1′′), 7.95 (t, *J* = 7.6 Hz, 1H, H-5N), 8.13 (s, 1H, H8), 8.63 (d, *J* = 7.6 Hz, 1H, H-4N), 8.96 (d, *J *= 6.11 Hz, 1H, H-6N), 9.07 (s, 1H, H2), 9.16 (s, 1H, H-2N). 

^13^C-NMR (100 MHz, D_2_O-*d*_6_): *δ* 8.6, 20.2, 45.7, 65.2, 65.5, 70.4, 74.6, 79.1, 79.9, 80.3, 86.5, 100.1, 100.3, 119.8, 125.6, 130.4, 135.8, 140.1, 143.8, 149.6, 152.7, 152.9, 156.4, 168.3.

^31^P-NMR (D_2_O, 121 MHz): *δ* −10.71 (m, 2P, P-O-P).

HRMS (ESI−) calcd for C_22_H_29_N_7_O_14_P_2_ 677.1127 (M–Et_3_N–H)^−^, found 677.1130.

#### 3.1.3. P^1^-[5′-(3′-*C*-Methyl-β-D-ribofuranosyl)adenine]-P^2^-[5′-(β-D-ribofuranosyl) nicotinamide] Pyrophospshate (Triethylammonium Salt) (3′-MeNAD, **2**)

In a similar manner as reported for compound **1**, coupling of imidazolide **9** (35 mg, 0.103 mmol) with monucleotide **6** (58.6 mg, 0.162 mmol) afforded pyrophosphate **2** (11.8 mg, 17% yield) as a white powder. Compound purity by analytical HPLC was 97% (t_R_ = 6.2 min).

^1^H-NMR (D_2_O, 500 MHz ): *δ* 1.21 (t, *J* = 7.3 Hz, 9H, (C*H*_3_CH_2_)_3_NH^+^), 1.45 (s, 3H, CH_3_), 3.05 (q, *J* = 7.1 Hz, 6H, (CH_3_C*H*_2_)_3_NH^+^), 4.11–4.48 (m, 7H, H3′′, H4′, H4′′, H5′, H5′′), 4.78 (t, *J* = 5.6 Hz, 1H, H2′′), 5.31 (d, *J* = 7.5 Hz, 1H, H2′), 6.04 (s, 1H, H1′′), 6.11 (d, *J* = 7.1 Hz, 1H, H1′), 8.17 (t, *J* = 7.6 Hz, 1H, H-5N), 8.31 (s, 1H, H8), 8.81 (d, *J* = 8.42 Hz, 1H, H-4N), 9.08 (d, *J* = 6.6 Hz, 1H, H-6N), 9.17 (s, 1H, H-2N), 9.31 (s, 1H, H2). 

^13^C-NMR (100 MHz, D_2_O-*d*_6_): *δ* 8.5, 16.6, 45.6, 65.1, 65.8, 70.8, 74.2, 75.7, 77.9, 83.3, 86.6, 96.5, 100.1, 119.4, 125.1, 130.7, 135.3, 140.3, 143.8, 149.8, 152.4, 152.6, 156.1, 168.1.

^31^P-NMR (D_2_O, 121 MHz): *δ* −11.044 (brs, 2P, P-O-P).

HRMS (ESI−) calcd for C_22_H_29_N_7_O_14_P_2_ 677.1127 (M–Et_3_N–H)^−^, found 677.1131.

#### 3.1.4. P^1^-[5′-(2′-*C*-Methyl-β-D-ribofuranosyl)adenine]-P^2^-[5′-(β-D-ribofuranosyl) nicotinic acid] Pyrophospshate (Triethylammonium Salt) (2′-MeNaAD, **3**)

In a similar manner as reported for compound **1**, coupling of β-NaMN imidazolide **10** (25 mg, 0.074 mmol) with monucleotide **5** (29.6 mg, 0.16 mmol) gave pyrophosphate **3** (16.4 mg, 25% yield) as a white powder. Compound purity by analytical HPLC was 98% (t_R_ = 6.3 min).

^1^H-NMR (D_2_O, 500 MHz): *δ* 0.82 (s, 3H, CH_3_), 1.15 (t, *J* = 7.3 Hz, 9H, (C*H*_3_CH_2_)_3_NH^+^), 3.13 (q, *J* = 7.2 Hz, 6H, (CH_3_C*H*_2_)_3_NH^+^), 4.08–4.21 (m, 4H, H5′, H5′′), 4.27–4.38 (m, 2H, H4′, H4′′), 4.41 (d, *J* = 7.6 Hz, 1H, H3′), 4.47 (t, *J* = 6.6 Hz, 1H, H3′′), 4.75 (pseudo t, 1H, H2′′), 5.84 (brs, 1H, H1′), 5.96 (d, *J* = 5.4 Hz, 1H, H1′′), 7.55 (t, *J* = 7.1 Hz, 1H, H-5N), 8.11 (d, *J* = 7.6 Hz, 1H, H-4N), 8.22 (s, 1H, H8), 8.81 (d, *J* = 6.4 Hz, 1H, H-6N), 8.96 (s, 1H, H2), 9.06 (s, 1H, H-2N).

^13^C-NMR (100 MHz, D_2_O-*d*_6_): *δ* 8.7, 20.1, 45.7, 65.3, 65.7, 70.8, 74.2, 79.4, 79.8, 80.5, 86.7, 100.1, 100.5, 119.7, 123.8, 127.9, 138.2, 140.3, 148.3, 149.8, 149.6, 152.4, 156.1, 166.3.

^31^P-NMR (D_2_O, 121 MHz): *δ* −10.8 (q, *J_αβ_*, *J_βα_* = 20.15 Hz, 2P, P-O-P).

HRMS (ESI−) calcd for C_22_H_28_N_6_O_15_P_2_ 678.1088 (M–Et_3_N–H)^−^, found 678.1090.

#### 3.1.5. P^1^-[5′-(3′-*C*-methyl-β-D-ribofuranosyl)adenine]-P^2^-[5′-(β-D-ribofuranosyl) Nicotinic acid] Pyrophospshate (Triethylammonium Salt) (3′-MeNaAD, **4**)

In a similar manner as reported for compound **1**, coupling of imidazolide **10** (25 mg, 0.074 mmol) with monucleotide **6** (29.5 mg, 0.081 mmol) afforded pyrophosphate **4** (15.8 mg, 24% yield) as a white powder. Compound purity by analytical HPLC was 98% (t_R_ = 6.1 min).

^1^H-NMR (D_2_O, 500 MHz): *δ* 1.15 (t, *J* = 7.3 Hz, 9H, (C*H*_3_CH_2_)_3_NH^+^), 1.35 (s, 3H, CH_3_), 2.83 (q, *J* = 7.2 Hz, 6H, (CH_3_C*H*_2_)_3_NH^+^), 3.96–4.38 (m, 7H, H3′′, H4′, H4′′, H5′, H5′′), 4.98 (t, *J* = 5.4 Hz, 1H, H2′′), 5.43 (d, *J* = 7.1 Hz, 1H, H2′), 6.01 (s, 1H, H1′′), 6.07 (d, *J* = 6.6 Hz, 1H, H1′), 7.82 (t, *J* = 7.1 Hz, 1H, H-5N), 8.37 (s, 1H, H8), 8.55 (d, *J* = 7.9 Hz, 1H, H-4N), 8.76 (d, *J* = 6.1 Hz, 1H, H-6N), 8.86 (s, 1H, H-2N), 8.96 (s, 1H, H2). 

^13^C-NMR (100 MHz, D_2_O-*d*_6_): *δ* 8.6, 16.7, 45.6, 65.2, 65.6, 70.4, 74.5, 76.1, 77.9, 84.1, 86.8, 77.9, 96.4, 100.5, 119.5, 123.8, 130.7, 127.9, 138.2, 140.5, 148.3, 149.7, 156.1, 166.3.

^31^P-NMR (D_2_O, 121 MHz): *δ* −11.06 (q, *J_αβ_*, *J_βα_* = 20.20 Hz, 2P, P-O-P). 

HRMS (ESI−) calcd for C_22_H_28_N_6_O_15_P_2_ 678.1088 (M–Et_3_N–H)*^−^*, found 678.1091.

#### 3.1.6. 9*H*-(2′-*C*-Methyl-β-D-ribofuranosyl)adenine-5′-monophosphate Tributyl Ammonium Salt (**5**)

A solution of phosphorous oxychloride (0.96 mL, 16.4 mmol) in triethyl phosphate (5 mL, precooled with ice-water) was added dropwise to a solution of compound **11** (1.4 g, 4.97 mmol) in trimethyl phosphate (31.5 mL) at 0 °C. The resulting solution was kept at 0 °C for 17 h. The mixture was then added to a solution of TEAB (0.5 M, 20 mL, cooled with ice water). After stirring for 10 min, the mixture was extracted with CHCl_3_ (3 × 100 mL). The aqueous layer was concentrated, and the residue was dissolved in water and purified by chromatography on a DEAE Sephadex (HCO_3_^−^ form) column, eluting with a linear gradient of H_2_O and 0.5 M NH_4_HCO_3_. The appropriate fractions were pooled and evaporated to dryness, obtaining an amorphous solid. This compound was converted into its free acid form by passing it through a Dowex 50 × 8 (H^+^) column, eluting with water, and then dried in vacuo in the presence of P_2_O_5_ overnight (1.18 g, 66%). It was suspended in dry DMF (20 mL), and (*n*-Bu)_3_N (1.1 mL, 1.87 mmol) was added. The mixture was stirred for 30 min. at room temperature and then concentrated under vacuum to obtain **5** as a white powder (63% yield).

^1^H-NMR (500 MHz, D_2_O-*d*_6_): *δ* 0.85 (s, 3H, CH_3_), 0.95 (t, *J* = 7.1 Hz, 9H, (C*H*_3_CH_2_CH_2_CH_2_)_3_NH^+^), 1.32 (m, 6H, (CH_3_C*H*_2_CH_2_CH_2_)_3_NH^+^), 1.72 (m, 6H, (CH_3_CH_2_C*H*_2_CH_2_)_3_NH^+^), 3.23 (t, *J* = 7.2 Hz, 6H, (CH_3_CH_2_CH_2_C*H*_2_)_3_NH^+^), 4.10–4.15 (m, 2H, H5′), 4.21 (pseudo q, 1H, H4′), 4.31 (d, *J* = 7.7 Hz, 1H, H3′), 6.15 (s, 1H, H1′), 8.35 (s, 1H, H8), 8.55 (s, 1H, H2). 

^13^C-NMR (100 MHz, D_2_O-*d*_6_): *δ* 13.7, 18.5, 20.4, 25.4, 54.5, 75.1, 79.3, 79.8, 80.6, 100.5, 120.3, 140.4, 149.8, 152.4, 156.3.

^31^P-NMR (121 MHz, D_2_O): *δ* 0.79 (brs, 1P, P-O). 

HRMS (ESI−) calcd for C_11_H_15_N_5_O_7_P 360.1465 (M–H)^−^, found 360.1452.

#### 3.1.7. 9*H*-(3′-*C*-Methyl-β-D-ribofuranosyl)adenine-5′-monophosphate Tributyl Ammonium Salt (**6**)

In a similar manner as reported for compound **5,** phosphorylation of nucleoside **12** (450 mg, 1.65 mmol) afforded nucleotide **6** as a white powder (reaction time 12 h, 63% yield). 

^1^H-NMR (500 MHz, D_2_O-*d*_6_): *δ* 0.92 (t, *J* = 7.1 Hz, 9H, (C*H*_3_CH_2_CH_2_CH_2_)_3_NH^+^), 1.33 (s, 3H, CH_3_), 1.38 (m, 6H, (CH_3_C*H*_2_CH_2_CH_2_)_3_NH^+^), 1.74 (m, 6H, (CH_3_CH_2_C*H*_2_CH_2_)_3_NH^+^), 3.19 (t, *J* = 7.2 Hz, 6H, (CH_3_CH_2_CH_2_C*H*_2_)_3_NH^+^), 3.92–4.03 (m, 2H, H5′), 4.20 (pseudo q, 1H, H4′), 4.50 (d, *J* = 7.9 Hz, 1H, H2′), 6.02 (d, *J* = 7.6 Hz, 1H, H1′), 8.22 (s, 1H, H8), 8.55 (s, 1H, H2). 

^13^C-NMR (100 MHz, D_2_O-*d*_6_): *δ* 13.6, 16.6, 18.7, 25.6, 54.4, 72.2, 72.9, 75.7, 83.3, 96.5, 119.1, 140.3, 149.5, 152.1, 156.4. 

^31^P-NMR (D_2_O, 121 MHz): *δ* 0.79 (s, 1P). 

HRMS (ESI−) calcd for C_11_H_15_N_5_O_7_P 360.1465 (M–H)^−^, found 360.1459.

### 3.2. Biochemical and Computational Methods

#### 3.2.1. NMN Adenylyltransferase Activity Assays

The reaction catalyzed by NMN adenylyltransferase can be represented as NMN + ATP (NAD + PPi). To avoid interference of methyl NAD analogues with NAD-based detection assays, we used a “pyrophosphate-dependent” coupled assay that couples NMNAT activity with inorganic pyrophosphatase (PPase) that hydrolyzes PPi into two molecules of inorganic phosphate (Pi). Phosphate was quantified using a malachite green dye as described [35]. Enzyme purification procedures followed previous reports [12]. The reactions were carried out using a 96-well microplate assay format at room temperature in a total volume of 50 µL per well. The reaction mixtures contained 100 mM Hepes, pH 7.5, 10 mM MgCl_2_, 0.1 mg/mL bovine serum albumin, and an excess of 4 U/mL of inorganic pyrophosphatase. The *h*NMNAT concentration, ranging from 3 to 15 nM, was adjusted in different tests to ensure initial velocity conditions (5–10% substrate depletion) while retaining a linear response. An amount of 100 µL of the malachite green reagent was added to each well to stop the reactions. Following a 20–30 min incubation period, the molybdate in the reagent formed a complex with the free phosphate, resulting in full-color development. The phosphate concentration was then quantified by measuring the absorbance at 620 nm using a microplate reader (Biotek Synergy HT) and interpolating these values against a pre-established standard curve. Absorbance from control reactions performed in the absence of *h*NMNAT were subtracted from absorbances of the respective reactions with the enzyme to account for background hydrolysis. Details for substrate and inhibitor concentration range are given below and in the legend of Figure 2. Assays with the inhibitors were carried out in triplicate, and the percentage of inhibition at each concentration was calculated for the initial screening at 0.5 mM.

For *K*_i_ determination, the enzyme was preincubated with various fixed concentrations of inhibitors for 5 min. The reaction was initiated by adding a fixed concentration of NMN (0.5 mM) at varying concentrations of ATP (centered at *K*_m_ value) and vice versa. Values for inhibition constant *K*_i_ and α were determined by fitting the data to the rate equation for general mixed model inhibition [36]: V = V_max_S/[*K*_m_(1 + I/*K*_i_) + S(1 + I/αI)] using GraphPad Prism software version 7.05, where V_max_ and *K*_m_ are the classic Michaelis–Menten parameters, *K*_i_ is the equilibrium dissociation constant for the enzyme-inhibitor complex, S and I are the substrate and the inhibitor concentration, respectively. This model includes competitive, uncompetitive, and noncompetitive inhibition as special cases that the α values can diagnose. Further mechanistic insights into the mode of inhibition were drawn by graphical inspection of the Lineaweaver–Burk plots.

#### 3.2.2. Protein Structural Modeling

A structural model of *h*NMNAT-2 was obtained with a homology modeling approach in 2010 by Brunetti et al. [37]. Recently, neural network-based methods for protein structure prediction have outperformed homology modeling programs [38,39]. Consequently, we produced a high-quality model of *h*NMNAT-2 (Uniprot ID Q9BZQ4) with AlphaFold2 deep learning method [38]. In particular, we ran the fast and more accessible ColabFold 1.5.5 software [40] at Google Colaboratory on an A100 GPU. Due to the intrinsic disorder of the central noncatalytic region of *h*NMNAT-2, which is responsible for its subcellular location to the Golgi apparatus [41], we excluded this part for structural modeling. To maximize accuracy, we increased the recycling parameter to 10, which decreased the uncertainty compared to the three default recycles. The predicted structures were energy-minimized, and the best model out of five was selected for further analysis. A predicted full-length model deposited in AlphaFold DB (https://alphafold.ebi.ac.uk/entry/Q9BZQ4, accessed on 5 April 2024) is also present.

#### 3.2.3. Flexible Docking, Sequence, and Structural Analysis

Molsoft ICM-Pro version 3.9 was used for protein display and superposition, in silico flexible docking, and analysis of ligand-pocket interactions [42]. The model of *h*NMNAT-2 served as the template for the in silico binding analysis of 2′-MeNAD. The conformation observed in the crystal structure (or in models) is sometimes incompatible with the ligands. To minimize the odds of artifactual ligand–protein conformations, an induced fit (flexible) docking procedure was used to obtain a reliable pose in which most residues typically involved in the NAD binding site were set to be flexible. The structure was prepared with standard procedures, including optimizing the hydrogen bonding network and the orientation of His, Pro, Asn, Gln, and Cys residues. The structure-based multiple alignment was constructed using PROMALS3D, manually edited and rendered with Jalview [43].

## 4. Conclusions

*h*NMNATs are recognized as significant drug targets for cancer chemotherapy, driving interest in developing high-affinity NMNAT inhibitors. Despite their established importance, potent and specific *h*NMNAT inhibitors remain scarce. This study evaluated a collection of 2′- and 3′-methyl-substituted NAD analogues (**1**–**4**) as potential inhibitors of *h*NMNAT isozymes. From this small structure–activity relationship study, 2′-MeNAD emerged as the most potent and selective *h*NMNAT-2 inhibitor reported to date, with K_i_ values towards NMN and ATP of 15 and 21 μM, respectively. Our results provided inhibition mechanistic insights that will be instrumental for future optimization of methyl NAD analogues: (a) the methylation at the adenosine moiety is effective only in the context of a complete NAD scaffold; (b) the distinct conformational preferences of 3′-MeNAD and 2′-MeNAD explain their striking differences in bioactivity; (c) an in silico analysis helped to dissect the 2′-MeNAD binding mode and 3D rationale of its bioactivity. Moreover, due to the universal conservation of the targeted adenosyl binding site across evolutionary distant members of the NMNAT family (Figure 3B), the 2′-methyl substitution at the adenosyl ribose could also fuel the ongoing exploration of anti-infective agents targeting NAD metabolism while minimizing the impact on the host as it does not seem to influence the ubiquitously expressed *h*NMNAT-1 and *h*NMNAT-3 forms. Future research trajectories should investigate potential off-target effects of these methyl NAD analogues to assess their toxicity or novel properties, given that hundreds of enzymes engage in the NAD scaffold. Additionally, evaluating more structural analogs that lack phosphate moieties should be pursued to overcome the membrane permeability limitations of these compounds.

## Figures and Tables

**Table 1:** Inhibition constant (*K*_i_) and mode of inhibition (in brackets) of the 2′-MeNAD/NaAD analogues on human NMNAT isozymes.

Product ^a^	*h*NMNAT-1		*h*NMNAT-2		*h*NMNAT-3	
	NMN	ATP	NMN	ATP	NMN	ATP
	*K*_i_ (μM) ^a^	*K*_i_ (μM)	*K*_i_ (μM)	*K*_i_ (μM)	*K*_i_ (μM)	*K*_i_ (μM)
2′-MeNAD (1)	N.D. ^b^	N.D.	15 ± 3 (M)	21 ± 3 (M)	312 ± 51 (C)	182 ± 53 (M)
2′-MeNaAD (3)	168 ± 19 (C)	82 ± 14 (U)	60 ± 18 (M)	41 ± 14 (U)	224 ± 17 (C)	223 ± 22 (C)

^a^ The inhibitory parameters of the two compounds were determined for the three human NMNAT isoforms by fitting the kinetic data to the general equation for the mixed or uncompetitive model of inhibition. The data were collected by varying the concentration of an inhibitor and one of the two substrates (NMN or ATP) at a fixed concentration of the other substrate. Inhibitors were tested at concentrations of 30 and 90 µM. *K*_i_ (or α*K*_i_ in the case of uncompetitive inhibition) values ± std. error are determined using nonlinear regression analysis as reported in the methods section. M, mixed inhibition; C, competitive; U, uncompetitive. ^b^ N.D., not determined, as the inhibition of *h*NMNAT-1 was below 50% at 0.5 mM inhibitor in the initial screening.

## Data Availability

Data are contained within the article.
